# Exploring Issues and Challenges of Leadership among Early Career Doctors in Nigeria Using a Mixed-Method Approach: CHARTING Study

**DOI:** 10.3390/ejihpe10010033

**Published:** 2020-02-14

**Authors:** Efosa Isibor, Kehinde Kanmodi, Oladimeji Adebayo, Olusegun Olaopa, Martin Igbokwe, Iyanu Adufe, Ibiyemi Oduyemi, Makinde Adebayo Adeniyi, Sebastine Oseghae Oiwoh, Ayanfe Omololu, Ifeanyichukwu Kelvin Egbuchulem, Selekeowei Peter Kespi Kpuduwei, Oluwafemi Efuntoye, Onuwabuchi Egwu, Oluwaseyi Ogunsuji, Elizabeth O. Grillo, Babalola Rereloluwa

**Affiliations:** 1 Irrua Specialist Teaching Hospital, Irrua 310112, Nigeria; 2 Kebbi Medical Centre, Kebbi 860223, Nigeria; 3 University College Hospital, Ibadan 200212, Nigeria; 4 Obafemi Awolowo Teaching Hospital, Complex, Ile Ife 17153, Nigeria; 5 University of Ibadan, Ibadan 200211, Nigeria; 6 Federal Teaching Hospital, Ido Ekiti 360211, Nigeria; 7 LAUTECH Teaching Hospital, Ogbomoso 210271, Nigeria; 8 Federal Medical Centre, Abeokuta 110242, Nigeria; 9 Federal Teaching Hospital, Gombe 771104, Nigeria

**Keywords:** leadership behaviour, challenges, issues, early-career doctors, clinical, Nigeria, organizational psychology, organisation

## Abstract

(1) Background: leadership behaviour is a poorly explored phenomenon among early-career doctors (ECDs). Good leadership is vital in maximising the effective management of patients in a clinical setting. While a good number of studies, though with small sample surveys, have researched the role of leadership in clinical setting quantitatively, qualitative investigations are yet to be done in Nigeria. This study aims to explore the attitudes, skills, and experience of ECDs in Nigeria on issues pertaining to leadership in a medical setting, using a mixed-method approach. (2) Methods: we conducted two sessions of key informant focus group discussion (FGD) that involved 14 ECD leaders in Nigeria, exploring their leadership experience in a clinical setting. Furthermore, we used a self-administered questionnaire to quantitatively survey 474 ECDs from seven Nigerian teaching hospitals to explore their attitudes, skills, and experience on issues pertaining to medical leadership. (3) Results: taking on leadership roles is a common phenomenon (52.7%) among the surveyed ECDs; however, the medical leadership position can be very challenging for ECDs in Nigeria. Despite the fact that many (91.1%) of the surveyed ECDs perceived leadership skills as essential skills needed by a doctr, many (44.1%) of them were yet to be formally trained on medical leadership. About three out of every 10 (23.6%) of surveyed ECDs that have ever held leadership positions in a medical setting experienced major leadership challenges while in such office due to their lack of training on leadership skills. Leadership skill acquisition programmes are highly recommended to become an integral part of medical training programmes in Nigeria. (4) Conclusion: there is a need for a structured leadership skill acquisition programme for ECDs in Nigeria. This programme will help in the robust delivery of highly effective healthcare services in Nigeria, as effective leadership is crucial to patient care services.

## 1. Introduction

Leaders are individuals of influence who take utmost responsibility for the progress of an organization [1]. They pilot the activities of an organization, show the way and inspire meaningful growth via their personality, sound principles and effective strategies [1]. The role of the physician has been expanded to involve managerial and leadership roles. Furthermore, health care service delivery revolves around teamwork, and it requires effective and high-quality leadership to improve team dynamics, produce enviable results and improve patient outcomes [2,3,4,5]. A doctor is required to lead multidisciplinary teams of health care professionals and to also exhibit a perfect blend of sound clinical acumen with excellent leadership and managerial skills [1,6].

Although doctors are understandably intelligent and well-read, due to the rigour of academic learning and clinical skill acquisition, this does not automatically translate into all doctors having sound leadership skills [1,7]. Medical school education is almost exclusively focused on the acquisition of skills needed in solving the clinical aspects of patients’ problems, with little to no attention paid to the need for medical students to acquire medical leadership skills [1,7]. Generally, until recently, leadership skill training/acquisition among doctors-in-training in Nigeria was usually postponed until the latter years of postgraduate (specialty) training—a period when most of them are also occupied with issues pertaining to their dissertation write-ups, and how they can acquire a good mastery of advanced specialist skills [1,8].

The fact that the qualities of a good doctor go far beyond just having sound clinical acumen cannot be overemphasised. There is a growing need for doctors to display good leadership qualities. These skills include proper communication; understanding of team personalities and dynamics; respect for other professional colleagues (senior, junior and even non-medical colleagues); responsible behaviour and proper accountability, and more [4]. Being the profession with the most expansive knowledge about patient care, doctors have the biggest legal responsibility to patients. This naturally thrusts the doctor into the forefront of the clinical team and makes it imperative for them to organise and coordinate the activities of the team. Although there are controversies at times amongst certain quarters about who is the best leader of the team, the available research has shown that the doctor is still best positioned to occupy this role [9].

All health systems throughout the world have their challenges, and the Nigerian system is not left out. Inadequate funding, loss of workers’ interest and job dissatisfaction are among some of the numerous problems bedevilling our system [1]. Anyaehie et al. have stated, amidst other issues, that limited or inexistent trainer–trainee interaction opportunities; frequent berating of doctors-in-training by supervising doctors; and limited leadership training opportunities for doctors-in-training were significant barriers to leadership skill acquisition among doctors-in-training in Nigeria [1,10].

Early career doctors (ECDs) are qualified medical practitioners who have finished first degree training and at least have certification to practice clinically and they include pre-registration house officers, medical/dental officers below the rank of a principal medical officer or its equivalent and resident doctors in Nigeria [11]. They are usually about ten years post primary medical and dental qualification [12,13]. Pertinently, there exists a paucity of research works of literature exploring the attitudes, skills, and experience of early career doctors (ECDs) in Nigeria on leadership issues in a medical setting. This shows that there is an imminent need to conduct a scientific study, preferably a large multi-centre study, to explore these underexplored areas of interest among ECDs in Nigeria. In order to achieve an in-depth exploration of these underexplored research themes (i.e., attitudes, skills, and experiences of early career doctors (ECDs) in Nigeria on issues pertaining to leadership in a medical setting), a mixed-method (i.e., a combination of qualitative and quantitative methods) approach is highly recommended.

Based on the above, we conducted this study with the aim of profoundly exploring the attitudes, skills, and experience of early career doctors (ECDs) in Nigeria on issues pertaining to leadership in a medical setting, using a mixed-method approach.

## 2. Materials and Methods

### 2.1. Nature of the Study

This study adopted the use of a mixed research method approach. This study also forms a part of the bigger and ongoing CHARTING study among ECDs participants in Nigeria [11,12,13]. Two focus group discussion (FGD) sessions involving a total of 14 participants were conducted to obtain qualitative data, while a sample of 474 ECDs in seven Nigerian hospitals was surveyed using a self-administered paper questionnaire to obtain quantitative data on issues about leadership among them. The participants recruited for this study were drawn from hospitals domiciled in South-West (SW), South-South (SS), North-Central (NC), and North-West (NW) geo-political zones in Nigeria.

### 2.2. Data Collection

#### 2.2.1. FGD

Two separate FGDs were conducted during two separate regular national meetings of the Nigeria Association of Resident Doctors (NARD), where key executives (such as national executive committee, national executive council, and expanded national executive council) and non-executive members of each state branch of the association came around for the meeting.

Participants’ recruitment into the study was voluntary, as only those participants who indicated their interest were allowed to partake in the study, although the majority of the participants were ECD leaders. We employed two trained facilitators to collect data, and the two FGD sessions lasted, on average, 1 hour 15 minutes. The sessions were not prolonged so as to prevent exhaustion among participants. The facilitators used a semi-structured FGD guide (see Appendix A) which was carefully designed to encourage the participants to express themselves on issues pertaining to their leadership experience in a medical setting.

The discussions were digitally recorded with the use of an audio recorder (Sony ICD-PX470 digital voice recorder), while a smartphone audio recorder was used as a backup/alternate plan, with participants consenting to ensure that the details of the conversations were adequately captured. Two sessions were conducted until data saturation was achieved (i.e., repetitive responses and lack of new information).

#### 2.2.2. Questionnaire Survey

The participants were administered structured questionnaires which obtained their basic socio-demographic data and information regarding attitude, perception and experience of leadership. This questionnaire had also been previously described in the study protocol [12].

### 2.3. Analysis

#### 2.3.1. FGD

Audio recordings were transcribed verbatim. Transcripts were analysed and thematically coded according to the research themes that emerged from the discussions. Coding was done using the NVivo 12 program. Open coding was also used to identify specific themes that emerged from the discussions. Themes and subthemes were generated and supported with illustrative quotations from the discussion.

#### 2.3.2. Questionnaire Survey

All collected questionnaires, which were in the English language, were subjected to screening for possible discarding if any of them were found improperly or incompletely filled out. Data were coded and computed into the Statistical Package for Social Sciences (SPSS) version 23 software for analysis. Data cleaning was also conducted immediately after data computation, before the data analysis was done. The frequency distributions of all variables were determined. Moreover, the Chi-square test was used to test for associations between qualitative variables; a p-value of < 0.05 was considered statistically significant. Generated results were depicted in words, charts and tables.

### 2.4. Ethical Considerations

This study was conducted under strict compliance with the 1964 Helsinki Declaration. Ethical clearance to conduct this study was obtained from the National Ethics Review Committee, Federal Ministry of Health (FMoH) Nigeria (NHREC Approval Number: NHREC/01/01/2007–26/06/2019). Written and verbal informed consent was obtained from all participants before their participation in the study. All information obtained from each participant, including personal details, was treated with the utmost confidentiality. To ensure privacy and confidentiality, all participants in the FGD were given an identifier, and anonymous questionnaires were used in the survey.

## 3. Results

### 3.1. FGD

#### Socio-Demographic Data of Respondents

The majority (85.7% (12)) of the respondents were male, while 14.3% (two) were females. Eight respondents were from SW (57.2%), while six respondents were from SS (42.8%). The distribution of the respondents was rich: two respondents were from University College Hospital, Ibadan (UCH), two were from Obafemi Awolowo University Teaching Hospital (OAUTH), two were from Lagos University Teaching Hospital (LUTH), two were from LAUTECH Teaching Hospital (LTH), two were from Rivers State University Teaching Hospital (RSUTH), three were from the Federal Medical Centre (FMC), Yenagoa, and one was from Niger Delta University Teaching Hospital (NDUTH).

Based on professional status, one respondent was a house officer, one was a senior medical officer, four were registrars, and eight were senior registrars.

### 3.2. Thematic Findings

The results of our qualitative data analysis show that effective clinical leadership is associated with optimal hospital performance. Four themes were identified which represent perception, experience, types of leadership skills and recommendation. These themes were presented with supportive quotes to buttress respondents’ experiences further.

(1) General View/Perceived Attitude Towards Acquiring Leadership Skills

As observed in this study, variations existed in respondents’ views on leadership. For some, it is an inborn quality and sometimes a bestowed responsibility; for others, it is the passion to make a notable change. Thus, the understanding of leadership is that it is about making an impact and also contributing to the system. The below transcripts illustrate the described analogies.

#### 3.2.1. Inborn Quality and Bestowed Responsibility

*“There is this saying that it is either you are born great, you achieve greatness or greatness was entrusted on you so for some of us greatness or been leaders was entrusted on some of us, we had no choice in the matter” (R7).*

#### 3.2.2. Passion to Make a Notable Change

*“I will say it is a passion to see things change, I will call it in summary a paradigm shift. It is better that you are an actor than you are actually watching the screen” (R5).*

*“For elective leadership positions personally just like every other member I discovered that prior to when I started aspiring for positions, I once looked at my leaders and discovered that they’ve not been doing enough and so there was this drive that ok if sometimes if things are glaring that needs change but you discover that the change is not coming so there is this drive probably if you are viewing it in this way why not take up the position and effect that change. So the desire for change drives for obtaining a leadership elective positions” (R3).*

*“The practicality of leadership actually comes when you are immersed in the leadership role itself, and that comes better when you are a senior registrar, or you are a consultant" (R8).*

*“The yearning for you to be a leader is to make an impact; impact in your association, impact in the wider body of NARD, impact even in your department” (R1).*

*“The reason why we want to go into this leadership position is because we are sick and tired of other people making the laws for us" "People that understand the intricacies of medical ethics should go in there and effect the change, and those people that are outside will come back to this country that is what I glamour for” (R5).*

(2) Experience of Leadership Positions in Medical and Clinical Settings

For many respondents, leadership positions in medical settings was described as challenging experience, but the main goal of leadership was to make an impact. The below statements justify these proclamations.

#### 3.2.3. Challenging Experiences

*“I think leadership role in this environment is not easy because it is like a car that is not working and you are trying to jump-start so you tend to put in a lot of energy to drive that car, so you tend to burn out a lot, so it is not easy been a leader in a system that is not working because you tend to put in a lot much more to see things work. It is quite challenging I must say” (R5).*

*“I remember in my department while we started having good pass rate was when our chief resident stood that no (no-no) when you are doing exams don’t come to the clinic and the consultant fought, and she stood her ground that it’s not happening and after that our result dramatically changed and when the consultants saw that the result has changed they backed down, so that is the foremost is to make impact that is why we are going for leadership positions” (R1).*

*“I’m part of the welfare unit in my department, and over time we found out that all we do is contribute into the welfare purse without been internalising it. We don’t feel the effect of our contribution. We found out that most of the things we do and the money we pay are been used for other things. We are using it for patient welfare like non-indigent patients, we are using it to repair this one and repair that one but into us as doctors we were not feeling the welfare. So we came together, and we told our HOD that we will not use our monies again, let’s use it for ourselves first. Our oath says we should first take care of our health. In taking care of ourselves and that drastically changed our work environment, you know when you are on calls you are calmer because the call rooms are better, well-equipped, ACs, refrigerators and all that. We did all that for our call rooms because definitely management does not go there, all they do is cleaning and they only most times focused on patient’s care, not on doctor’s care. So we had to take into consideration ourselves, and we made an impact. After that our calls has been better and our work experience even with the way we work changed because we were now taking care of ourselves, so that caused an impact in our output towards the patient” (R4).*

(3) Types of Leadership Skills Essential in Clinical Settings

Leadership skills that are vital in clinical settings are as follows: communication skills, listening skills, decision making skills, integrity, and being unbiased, as stated by the respondents:

#### 3.2.4. Communication Skills

*“First is communication, your ability to communicate not to order but to communicate. To be a communicator, to be able to pass across a message and the message is understood, and the instructions carried out. You are not an instructor, and you are not an enforcer" “Then you also have to have integrity in carrying out your own duties. Assuming you expect your residents to be at work at 7:30, you should be at work at 7:00 so when you show that form of integrity and diligence you are easily followed (you understand) for instance, we have a chief resident who…before you get there she is there, and she does not shout or scream or rain abuses or melt out punishment just because you know that she will get there before you, you have to get there before her (you understand) because that is her attitude of integrity, punctuality of being nice and humble so everybody started going that way” (R4).*

#### 3.2.5. Listening and Decision-Making Skills

*“A leader has to be a good listener” “A leader has to be a good decision maker because a lot of decision would impact on the welfare of the people the person so if he doesn’t know how to be a good decision maker it would impact negatively on the team” (R1).*

*“A leader has to be (how will I put it) firm when you make decisions you have to stick to them” (R1).*

#### 3.2.6. Integrity and Being Unbiased

*“A good leader in the health sector is to be professionally unbiased” “A good leader of a health sector, if you are a doctor and a leader you should know that you are not only handling the affairs of doctors; there are other professionals among them; the nurses are there, even the health attendants are there, so don’t be biased if a doctor is faulty; tell him and handle that situation as such not because you are a doctor and you start compromising your leadership skills it won’t augur well. So a good leader must be professionally unbiased, and that is the only way that other professionals in the health sector will come to respect that leader” (R5).*

(4). Recommendation

One respondent, however, recommended that leadership training should be formally integrated into the medical school curriculum.

#### 3.2.7. Curriculum Review

*“I think as part of our curriculum from the medical school; leadership should be inculcated because as medical doctors by virtue of that our position we are already leaders somehow (you know) and it is so bad that you will see doctors occupy leadership position and perform very woefully so I think it should be part of the curriculum while we are medical students they should be taught leadership so going forward when you become a doctor it will become part and parcel of you which you can always want to exercise good leadership skills (you know) that is my take on it” (R5).*

### 3.3. Questionnaire Survey

About one fifth (20.5%, 474/2317) of the ECDs of the seven selected hospitals in Nigeria were surveyed using a questionnaire. The majority (4/7) of these hospitals were located in the South-West geo-political zone of Nigeria. More than three quarters (83.6%) of the surveyed ECDs (respondents) were affiliated to hospitals in South-West Nigeria (Table 1).

The gender distribution of the respondents was skewed, with 67.3% of them being males. The majority (62.4%) of them were married, 36.1% were registrars, and their average (±SD) number of years spent in their current job position was 3.3 (± 2.7) years (Table 2).

**Figure 1:**
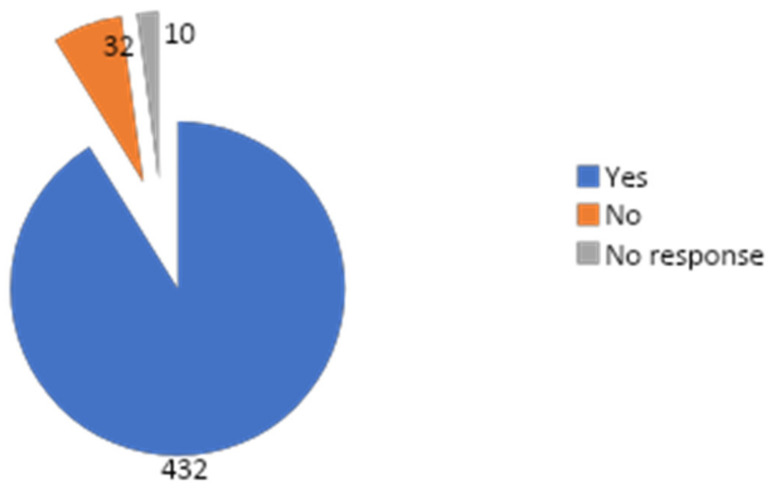
Distribution of responses of respondents to the question “Do you consider leadership and management skills important for doctors?”

**Figure 2:**
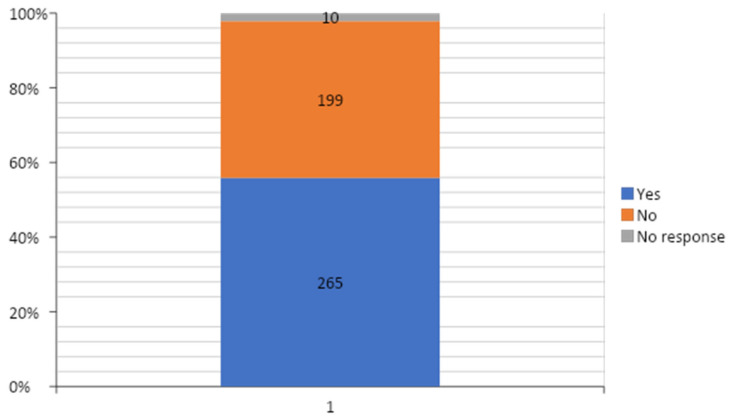
Distribution of responses of respondents to the question “Have you ever received leadership and management training?”

Four hundred and thirty-two (91.1%) respondents, comprised in the majority (36.3%) by junior registrars (Table 3), considered leadership and management skills as important skills to possess as a doctor (Figure 1). Only 265 (55.9%) respondents had ever received training on management and leadership (Figure 2) and the majority (38.1%, 101/265) of them received this training while in medical school (Table 4).

**Figure 3:**
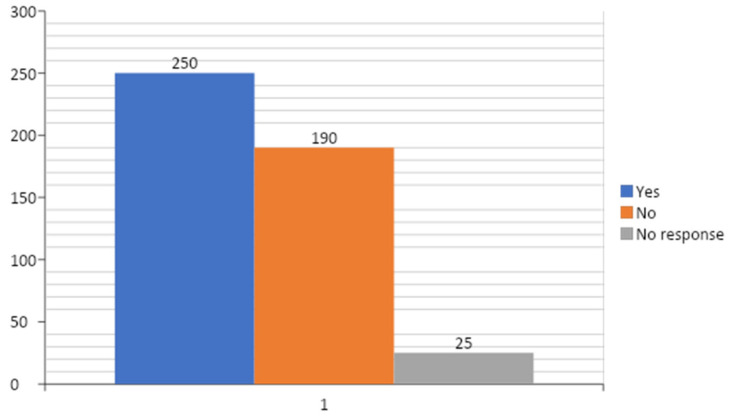
Distribution of responses of respondents to the question “Have you ever had the opportunity to assume a leadership role in your medical practice, so far?”

Two hundred and fifty (52.7%) respondents had been opportune in assuming leadership roles in their medical practice (Figure 3). The “lack of understanding from other members of the management team” was the most predominant challenge experienced by more than a third (36.8%) of those respondents who had ever held leadership positions in medical practice (Table 5); however, the top two most helpful sources of support when engaging in leadership and management roles, as indicated among them, were “senior colleagues” and “fellow trainees” (Table 6).

Lastly, from the bivariate analysis of the survey data, it was found that “respondents’ opinions on the cadre of doctors that require leadership and management skills”, “respondents’ opinions on the incorporation of leadership and management skill acquisition programmes into medical training programmes in Nigeria” and “respondents’ preferred source of acquiring leadership and management skills” had statistically significant relationships with “respondents consideration of leadership and management skills as an important skill for doctors” (p-values < 0.05) (Table 7).

## 4. Discussion

The 21st century doctor must, indeed, be well-grounded and trained to be an efficient administrator and manager in addition to their basic clinical responsibilities [3]. Effective clinical leadership is associated with optimal hospital performance. Effective clinical leadership is closely associated with a wide range of hospital functions, and it forms an integral component of the health care system. Unfortunately, poor administration and leadership has been persistently bedevilling the Nigerian health care system for many years, and it is the root cause of numerous industrial actions and unrest [14]. Developing clinical leadership skills among ECDs and other health professionals is of critical importance in changing the current trend. However, despite the widespread recognition of the importance of effective clinical leadership with regards to patient care, there are considerable barriers to participation in clinical leadership by ECDs [7]. Due to these barriers, only a fraction of ECDs in Nigeria have an interest in leadership positions [1,10]. To make things worse, not all ECDs have access to leadership training programmes in order to prepare them for the leadership requirements of the modern day.

It is noteworthy that a large number of doctors take on significant leadership responsibilities along the course of their career. The major challenges faced by ECDs, as found in this present study, were lack of training experience, lack of confidence, lack of support from fellow trainees, lack of support from senior doctors, lack of understanding from other members of the management team and lack of support from management. The core systemic solution from these itemised challenges is still adequate leadership skill acquisition training targeted at ECDs/medical students.

The postgraduate medical specialist training programme is heavily skewed towards the acquisition of knowledge and skills to manage clinical problems, with little attention towards formal and structured leadership and management skills training/acquisition [1]. Interestingly, most of the respondents believed that such training is necessary, and over half of them had undergone such training at some point in time. Those respondents who had received some form of leadership skill acquisition training may not be unconnected with the heterogeneous nature of their group in terms of their cadre and type. About a quarter of those respondents with a positive history of leadership skill acquisition were holding non-specialist training positions (such as house officers and medical officers) as at the time of their participation in this present study. It is, however, encouraging that a high proportion of the study respondents were exposed to leadership skill acquisition at the undergraduate level, contrary to held norms [1,2,8]. Probably due to their experience, many of those respondents with previous training experience on leadership skills believed that leadership and management training is necessary for doctors. Ojo et al., in their study, suggested the need to incorporate relevant training modules aimed at improving leadership skills among Nigerian doctors at both undergraduate and postgraduate levels by consistently featuring it in continuing medical education (CME) programmes [2]. They further encouraged more ECDs in Nigeria to pursue postgraduate degrees in Health Management Sciences. The leadership skills vital in clinical settings, as stated by Ojo et al., are communication skills, listening skills, decision making skills, integrity, etc. These aforementioned skills had also been described in the literature [1,4,6]. There may be a need to improve on the leadership and management training programme that doctors-in-training encounter during their postgraduate study programmes. Other suggested ways of building the leadership skills of ECDs include the incorporation of leadership training programmes at the undergraduate level; the encouragement of ECDs to enrol in masters degree programmes in medical leadership/health policy/health management, among others [4]. In addition, there may be a need to create professional bodies which are similar to the American College of Physician Executives for the provision physician/surgeon leadership training and accreditation in Nigeria [4].

Despite the fact that medical institutions have designated “leadership skill” as a core medical skill, leadership skills are still rarely taught and reinforced across the continuum of medical training. As more evidence shows that leadership skills and management practices positively influence both patient and healthcare organization outcomes, it is becoming clear that leadership training should be formally integrated into both undergraduate and postgraduate medical training curricula. There is also a need to emphasise multidisciplinary leadership training, not just for the ECDs but also for other health care professionals in order to make communication easier between these groups of workers [15]. A system should also be created to formulate structured training platforms/methods that encourage more trainer–trainee encounters. Structured training methods should include online lectures/webinars, short classroom lectures and role-playing methods, as popularly mentioned among the respondents. There is evidence that this helps develop leadership skills in clinical settings [16,17]. Leadership training for ECDs will further train them for middle-level management (such as leadership positions, like chief residents and heads of departments) and top-level management (such as leadership positions, like chief medical directors and ministers) [2,18]. This training exposure is also very crucial for those who intend to venture into private medical practice after completion of their postgraduate specialist training programmes. Furthermore, ECDs should be encouraged to take up leadership responsibilities while in that phase of their career. This would build the poor confidence identified in this study and also foster team building spirit.

The methodological limitation was recognised as only two geo-political zones were represented in the qualitatitive aspect of the study. However, the results of this study do make a valuable contribution to the knowledge of leadership and ECDs in Nigeria.

This is an under-researched area that requires further funding and focus. Based on our findings in the Nigerian health sector, coupled with the current state of the health care system in Nigeria, this will, significantly go a long way in providing guidance on the issues and challenges being experienced by ECDs in Nigeria, and invariably informing health policymakers on issues surrounding leadership roles among ECDs in Nigerian health care settings.

## 5. Conclusions

This study shows that leadership skills are very crucial skills needed for ECDs in Nigeria. The findings obtained in this study have generated important research questions that will hopefully serve as a stimulus for further studies into this subject, and will subsequently improve the leadership quality of ECDs in Nigeria. 

## Figures and Tables

**Table 1:** Distribution of target population and survey sample.

Centre	Geo-Political Zone	Target Population	Sample	% of n
FMC, Abeokuta	South-West	387	116	24.9
UCH, Ibadan	South-West	531	170	35.9
FTH, Ido Ekiti	South-West	194	72	15.2
JUTH, Jos	North-Central	504	17	3.6
FMC, Katsina	North-West	150	46	9.7
LAUTECH TH, Ogbomosho	South-West	89	36	7.6
UPTH, Port-Harcourt	South-South	460	17	3.6
Total	All zones	2315	474	100.0

Notes: n = total sample = 474.

**Table 2:** Basic demographic data of survey respondents.

Characteristics	N (%)
Sex	
Male	319 (67.3)
Female	155 (32.7)
No response	0 (0.0)
Age (Years)	
Mean (±SD)	33.5 (±5.7)
Marital Status	
Single	176 (36.1)
Married	296 (62.4)
Divorced	4 (0.8)
No response	3 (0.6)
Cadre	
House officer	109 (23.0)
Medical officer	37 (7.8)
Registrar	171 (36.1)
Senior registrar	146 (30.8)
No response	11 (2.3)
Number of Years after Bagging Medical/Dental Degree *	
Mean (±SD)	7.2 (±4.1)
Years of Practice	
Mean (±SD)	6.9 (±3.8)
Years Spent in Current Job Position	
Mean (±SD)	3.3 (±2.7)

Notes: SD = standard deviation; N = Number of respondents per category; *refers to MBBS/MBChB/MD/BDS/ BChD/DDS/DMD degree.

**Table 3:** Cadre distribution of respondents who considered leadership and management skills important for doctors.

Cadre	Frequency (%)
House officer	94 (21.8)
Medical officer	33 (7.6)
Junior registrar	157 (36.3)
Senior registrar	139 (32.2)
Unspecified	9 (2.1)
Total *	432 (100.0)

Notes: * Total number of respondents who considered leadership and management skills important for doctors.

**Table 4:** Period when those respondents with a history of leadership and management training experience received the training.

Period	Frequency (%)
In medical school	101 (38.1)
During internship	37 (13.9)
During junior residency	49 (18.5)
At senior residency	33 (12.5)
Others	32 (12.1)
No response	13 (4.9)
Total *	265 (100.0)

Notes: * Total number of respondents with positive history of receipt of leadership and management training

**Table 5:** Challenges experienced by those respondents with leadership experience in medical practice.

Challenges (N = 250)	Frequency (%)
Lack of training experience	59 (23.6)
Lack of confidence	23 (9.2)
Lack of support from fellow trainees	75 (30.0)
Lack of support from senior doctors	53 (21.2)
Lack of understanding from other members of the management team	92 (36.8)
Lack of support from management	51 (20.4)

Notes: N = Total number of respondents who had held leadership positions in medical practice.

**Table 6:** Most helpful sources of support when engaging in leadership and management roles indicated by those respondents who had held leadership positions in medical practice.

Source of Support	Frequency (%)
Management	37 (14.8)
Senior colleague(s)	80 (32.0)
Fellow trainee	82 (32.8)
Other members of the management team (non-doctors)	23 (9.2)

**Table 7:** Comparison between respondents’ perception of the importance of leadership and management skills with other factors.

	Do You Consider Leadership and Management Skills Important for Doctors? *				
		Yes	No	Total	p-value
Have you ever received leadership and management training? *	Yes	248	14	262	0.118
	No	180	18	198	
	Total	428	32	460	
Do you consider your medical training so far sufficient to carry out good leadership and management role? *	Yes	102	7	109	0.954
	No	314	21	335	
	Total	416	28	444	
Have you ever had the opportunity to assume the leadership role in your medical practice, so far? *	Yes	238	18	256	0.609
	No	178	11	189	
	Total	416	29	445	
What cadre of doctors require leadership and management skills? *	House officer	7	13	20	0.028
	Medical officer	4	2	6	
	Junior registrar	0	5	5	
	Senior registrar	0	6	6	
Have you ever been queried for exhibiting poor leadership and management skills? *	Yes	62	5	67	0.720
	No	328	22	350	
	Total	390	27	417	
Should leadership and management skill acquisition programmes be part of the medical training programme in Nigeria? *	Yes	408	25	433	<0.0001
	No	17	7	7	
	Total	425	32	32	
What is your preferred source of acquiring leadership and management skills? *	Rotations/outside postings for management courses	227	10	237	0.011
	At the yearly college update courses	67	11	78	
	At the daily departmental trainer–trainee encounter	118	9	127	
	Total	412	30	442	
What is your preferred mode of acquiring leadership and management skills? *	Online lectures/webinars	81	9	90	0.066
	Short classroom lectures	152	16	168	
	Case method	78	3	81	
	Role-playing method	104	3	107	
	Total	415	31	446	

Notes: * Only the data of those that responded to the cross-tabulated variables were shown in this table.

## Appendix A: Focus Group Discussion Guide

**S/No.**	**Questions**	**Probes**
1.	How would you describe residency training generally?	Who are those eligible for the training? Criteria used in selection? Views regarding residency duties? Do you think residency training helps with research?
2.	What do you think about leadership?	What is your motivation to take (non-elective) or contest (elective) for leadership position? Attitude and general disposition to leadership skills Experience and perception to leadership position in medical and clinical settings Types of leadership skills Frequency of training
3.	What are the challenges of leadership among ECDs?	
4.	What would you suggest or recommend to mitigate leadership challenge among early career doctors (ECDs)?	
